# Study of Tetrahydroxylated Anthraquinones—Potential Tool to Assess Degradation of Anthocyanins Rich Food

**DOI:** 10.3390/molecules26010002

**Published:** 2020-12-22

**Authors:** Lukáš Kučera, Ondřej Kurka, Martin Golec, Petr Bednář

**Affiliations:** 1 Department of Analytical Chemistry, Faculty of Science, Palacký University, 17. Listopadu 12, 779 00 Olomouc, Czech Republic; lukas.kucera@upol.cz (L.K.); ondrej.kurka@upol.cz (O.K.); 2 Department of History, Faculty of Arts, Palacký University, Křížkovského 10, 779 00 Olomouc, Czech Republic; martin.golec@upol.cz

**Keywords:** anthocyanin, degradation, hydroxyanthraquinone, phloroglucinaldehyde, wine

## Abstract

Degradation of anthocyanins involves scission of the flavonoid skeleton yielding 2,4,6-trihydroxybenzaldehyde (phloroglucinaldehyde, PGA) and a phenolic acid. However, the process is not finished with the formation of PGA, as the consequent condensation of two PGA molecules providing colored hydroxylated anthraquinones was observed for the first time. This process was studied using a combination of preparative column chromatography, nuclear magnetic resonance, liquid chromatography/high resolution tandem mass spectrometry (LC/HRMS^2^), and quantum calculations using density functional theory. 1,3,5,7-tetrahydroxyanthraquinone (anthrachrysone) and its isomers were found to rise during heating (95 °C) in a buffered PGA model solution (phosphate buffer, pH 7). These compounds were detected in heated red wine after an increase of its pH value. The concentration of the identified anthrachrysone in the red wine reached 0.01 mg·L^−1^. Presence of those compounds could therefore indicate involvement of certain steps in the processing of plant materials rich in anthocyanins (e.g., utilization of a higher temperature and/or reduction of acidity) or long-term transformation of anthocyanins (potentially, for instance, in archaeological findings such as wine or fruit residues). Additionally, measurement of wine–soil suspensions proved an increase of their pH to the values suitable for anthocyanin cleavage (neutral to slightly alkaline; reached using soil from archaeologically well-known Bull Rock Cave). Although not found in artificially prepared samples (imitations) or authentic materials so far, according to our results the above mentioned conditions are suitable for the formation of tetrahydroxylated anthraquinone derivatives and their monitoring would be beneficial.

## 1. Introduction

**Figure 1:**
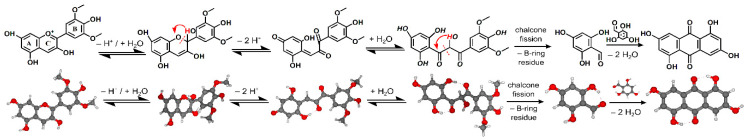
Proposed process of anthrachrysone formation from anthocyanin (malvidin) via phloroglucinaldehyde.

Anthocyanins are water soluble plant dyes present in a wide spectrum of fruits (e.g., red grapes, strawberries, and raspberries) in relatively high concentrations. For a long time, it has been known that those flavonoids possess many benefits for human health [1]. Because of their high contents in food prepared from plants and their wide use as natural food colorants, analysis of anthocyanins became a frequent part of food quality control. Stability of common anthocyanins is strongly influenced by many factors such as pH, temperature, concentration, presence of enzymes or bacteria, etc. [2,3]. Their degradation involves cleavage of the sugar moiety and scission of the heterocyclic ring in the flavonoid skeleton providing 2,4,6-trihydroxybenzaldehyde (phloroglucinaldehyde, PGA) on one side (bearing A-ring of the original anthocyanin) and a phenolic acid on the other (see the scheme in Figure 1). This process was observed in natural materials rich in anthocyanins such as blueberry pomace after its thermal hydrolysis and heating at pH 1, 4, and 7, respectively [4]. The structure of the phenolic acid is given by the substitution of B-ring of the original anthocyanidin, i.e., protocatechuic, *p*-hydroxybenzoic, 3,4-dihydroxy-5-methoxybenzoic, syringic, vanillic or gallic acid are formed [5]. Two molecules of phenolic acid can further mutually react and form tetrahydroxylated anthraquinones as already proven in model solutions [6,7]. Generally, the process of formation of hydroxylated anthraquinones occurs in nature, e.g., in the reaction of succinoylbenzoic acid, formed from shikimic acid and α-ketoglutaric acid, with mevalonic acid [8]. However, to the best of our knowledge analogous mutual condensation of two PGA molecules has not been reported until now and its examination is the task of this study. The process is shown in the last step of the scheme in Figure 1. The increased amount of PGA and phenolic acids and/or formation of hydroxylated anthraquinones can be in principle used to reveal former presence of anthocyanin dyes in a broad range of applications, e.g., food and forensic control or characterization of organic residues in archaeological and historical findings (solid remains of wine, musts, fruits, etc.). Note that phenolic compounds released from anthocyanins have high chemical and microbial stability that could be the reason for their long-term survival in organism (or environment) and the potential to inhibit oxidation stress during the whole time of their passing through digestive system (and also to affect intestinal microflora) [9,10].

Hydroxylated anthraquinones (HAQs) belong among natural dyes. Madder root (*Rubia tinctorum*) is a significant source of HAQs. More than seventy HAQs have been discovered in this plant material, but only a few of them are present in higher concentrations, i.e., alizarin, purpurin, danthron, and quinizarin [11]. Their effects on the human body have already been described [12,13,14,15,16]. For the identification of tetrahydroxyanthraquinones in extracts of metabolites of fungi *Geosmithia lavendula*, HPLC and UHPLC methods based on reversed phase stationary phases, photodiode array (PDA) detection, and off-line Fourier transformation ion cyclotron mass spectrometry (FT-ICR-MS) analyses were developed by Stodůlková et al. [17,18]. To the best of our knowledge, those two papers are the only studies devoted to the (U)HPLC/MS analysis of tetrahydroxylated anthraquinones. Although wider attention has not been paid to these substances so far, their analysis may be an interesting tool for assessing the degree of damage of anthocyanin-rich materials.

Processing of plant products rich in anthocyanins involves heating/boiling of plant material (hot maceration of grapes during wine production, fruit preserving, production of marmalades, etc.). It is also well known that the flavonoid skeleton is susceptible to scission at neutral and alkaline rather than in acidic conditions. Neutral to slightly alkaline conditions appear in some food treatments. For instance, black carrot flour was used as a replacement for wheat flour (healthy flour ersatz) for preparation of sponge cake in a recent study. At 8% substitution of wheat flour with black carrot flour, the pH of the material was neutral to slightly alkaline [19]. Moreover, acidity reduction cannot be precluded to occur when a food technology is used improperly. The combination of such conditions (pH ≥ 7 and heating) increases the speed of anthocyanin decomposition processes and could result in formation of tetrahydroxylated anthraquinones. Suitable conditions for anthocyanins decomposition and further mutual reaction of rising products can also be expected in the small intestine where neutral to slightly alkaline conditions are maintained for increasing nutrients’ bioavailability [20]

Heating speeds-up (to a certain extent) processes that take place over a very long time, i.e., those that could be expected in archaeological context (heating is one of the processes used during artificial aging of historically relevant materials [21]). Note that due to low stability of flavonoids, their degradation products can be easily identified in archaeological samples [22] while on the other hand anthraquinones proved to be more stable compounds as shown by Clementi et al. (these compounds including purpurin as the main anthraquinone colorant from madder did not degrade significantly during their accelerated aging when deposited on wool yarns) [23]. Contact of food products with soil and/or ceramic material (as often found during archaeological excavations) can cause a decrease of their acidity and, thus, support the processes leading to the scission of the anthocyanidin skeleton and the formation of PGA (pH of soil falls within the range of 6–8 [24]).

Recently, a number of analytical methods are used in polyphenols profiling. The most common technique for the analysis of anthocyanins and related phenolic compounds is liquid chromatography coupled to mass spectrometry [3,25,26,27,28]. It is the most advisable choice for investigating polyphenols due to high column capacity and robustness (compared for instance to capillary electrophoresis [29,30]). Besides, gas chromatography was used for analysis of flavonoids as well, however, it is limited by their lower volatility (and lower stability at higher temperatures in the same time) and there is often the necessity to involve a derivatization step in the sample preparation protocol. A comprehensive overview is given in the paper of Nolvachai and Marriott [31]. There are several standalone (without previous separation) spectral techniques used for the analysis of phenolic compounds in anthocyanin-rich foods, i.e., nuclear magnetic resonance [32], matrix assisted laser desorption/ionization–mass spectrometry [33], and infrared spectroscopy [34]. Those methods provide cumulative analytical information of all compounds in a complex matrix. However, serious matrix effects are commonly observed. Based on the overview of the pros and cons of the methods discussed above, LC/HRMS^2^ was selected as the main analytical tool in this study.

The contribution has two aims: (1) An exploration of the process of transformation of anthocyanins via their decomposition to phloroglucinaldehyde and its consequent condensation reactions and (2) A pilot LC/HRMS^2^ analysis of tetrahydroxyanthraquinones (THAQs) in red wine as the first step for the development of methods for food control and characterization of food residues originally rich in anthocyanins (in recent or archaeological context). Wine represents perhaps the most explored anthocyanins-rich food with a well-known polyphenol profile also formerly studied by our team [3,28].

## 2. Results and Discussion

**Figure 2:**
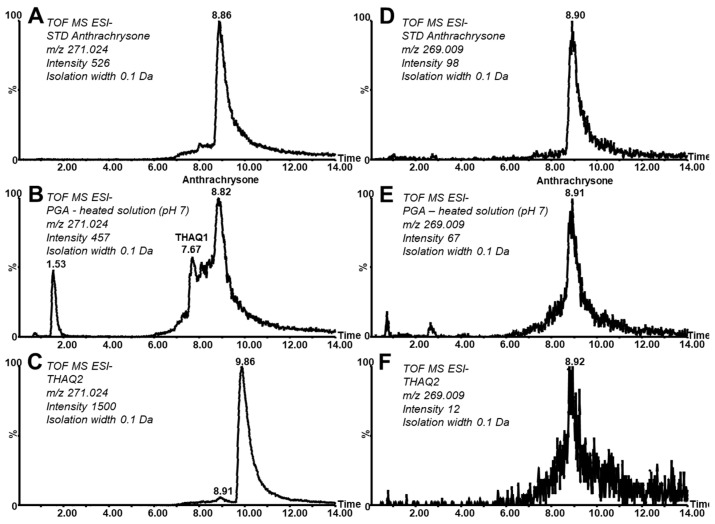
LC/MS analysis of anthrachrysone and its isomers. The reconstructed chromatograms at *m*/*z* 271.024: (**A**) standard of anthrachrysone (ATCS), (**B**) ATCS and its isomers formed in a heated solution of phloroglucinaldehyde (PGA), (**C**) tetrahydroxylated anthraquinone isomer 2 (THAQ2); and at *m*/*z* 269.009: (**D**) the oxidation form of ATCS in its standard solution, (**E**) the oxidation form of ATCS found in the heated solution of PGA, (**F**) THAQ2.

The first experiments were focused on the behavior of the standard of PGA in neutral buffered solution. Pronounced decomposition was observed (the effect of time and pH is discussed later). LC/HRMS^2^ analysis using Acquity UPLC H-Class combined with a Synapt G1 mass spectrometer revealed two newly formed compounds eluted at a retention time (RT) of 7.67 and 8.82 min, respectively, with parent ions recorded at *m*/*z* 271.0275 (both signals with identical mass) as can be seen in Figure 2B. The second peak at an RT of 8.82 min corresponds (considering its retention and *m*/*z* value) to the standard of 1,3,5,7-tetrahydroxyanthraquinone (known trivially as anthrachrysone, ATCS) prepared by an independent synthetic pathway and structurally confirmed by NMR (see Experimental). The deviation from the theoretical mass of elemental composition C_14_H_8_O_6_, dtm, is 3.2 mDa and the deviation of the *m*/*z* value from the *m*/*z* value of the standard is 0.0 mDa. Corresponding reconstructed chromatograms are given in Figure 2A.

**Figure 3:**
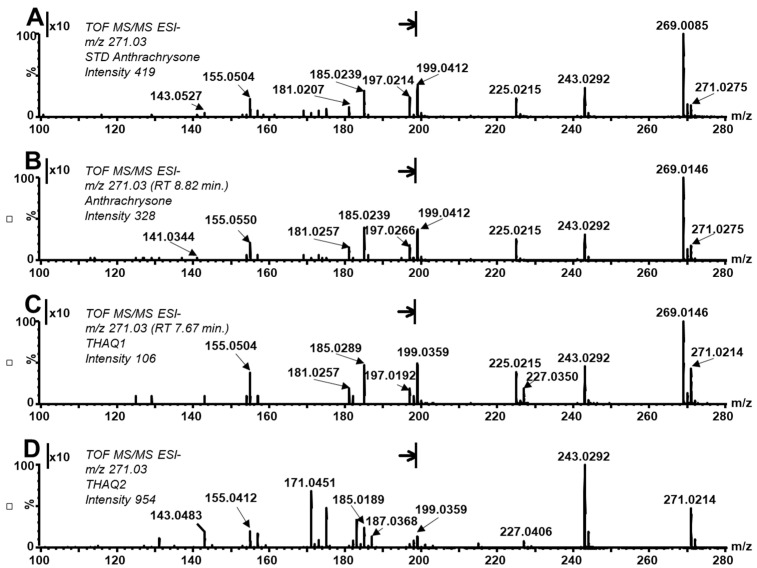
MS/MS spectra of ATCS standard (**A**), ATCS rising in a heated PGA solution (**B**), THAQ1 in the heated PGA solution (**C**), and THAQ2 (**D**). (*m*/*z* range 100–197, denoted by vertical lines and an arrow to the right, was zoomed 10 times).

Furthermore, the fragmentation pattern of the ATCS standard and the compound eluted at RT 8.82 min correspond well to each other (see MS/MS spectrum in Figure 3A,B), especially concerning fragments at *m*/*z* 243.0292, 225.0215, 199.0412, 185.0289, and 157.0276. The first two fragments arise by consecutive losses of carbon monoxide and water from the parent ion. The third one could be explained as a result of three consecutive processes: (1) cleavage of carbon monoxide (formation of fragment 243.0391), (2) tautomeric formation of 1,2-dioxolane ring by linkage of two oxygens bound at the carbon C1 and C9 (or C5 and C10) of the fragment, (3) excision of a carboxyl group (as carbon dioxide) from the central ring. The fragment at 185.0289 is proposed to be formed by the elimination of a neutral molecule with elemental composition C_3_H_2_O_2_ from one of the side rings (3-oxoacrylaldehyde or its isomers; Δ*m*/*z* 70) and a consequent loss of an oxygen radical (Δ*m*/*z* 16). The fragment at *m*/*z* 157.0276 rises by the scission of a CO molecule from the previously discussed fragment. Besides the above discussed fragments, ions at *m*/*z* 269.0085 are observed both in the MS/MS spectrum of the standard and in the studied compound eluted at RT 8.82 min. Since the studied hydroxylated anthraquinones are sensitive to oxidation at specific conditions, those signals were ascribed to the oxidation of the parent molecule (formation of [M−H-2H]^−^ ions not separated from the parent ions in the quadrupole during the MS/MS experiment). Such oxidation can occur during a heating experiment as well as, to a certain extent, during the sample preparation and in the autosampler before the injection. Some redox processes are described as occurring during the electrospray ionization and can change the ratio of the intensity of signals at *m*/*z* 271 and 269 as well. The main fragmentation processes, i.e., the loss of carbon monoxide and the excision of the carboxyl group, can occur from the parent ion as well as from its oxidized form and this fact significantly affects the appearance of the fragmentation spectra. The oxidized form of parent ion provides a characteristic and abundant fragment at 225.0215 (loss of CO_2_). Besides, fragments at *m*/*z* 241.0202 (loss of CO) and *m*/*z* 199.0412 (loss of CO + CO_2_) can be observed. Note, that the peak of oxidized ATCS elutes in the same RT as the parent molecule (Figure 2D,E) pointing to its formation in the ion source.

**Figure 4:**
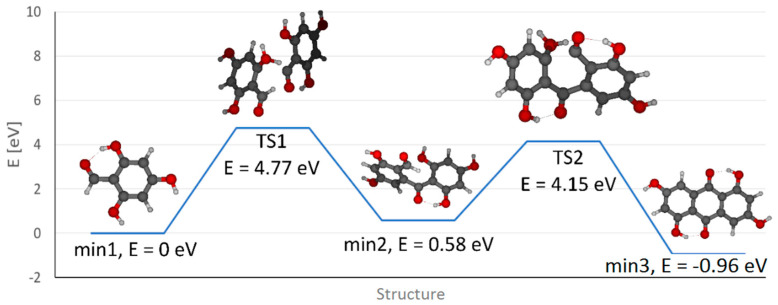
Energy profile of the phloroglucinaldehyde condensation to ATCS. (min—optimized structures with minimum energy; TS—transition states reflecting energetic barriers).

Moreover, theoretical quantum calculations also justify the appearance of ATCS. In our calculation, the process of condensation of two molecules of PGA was examined using density functional theory (DFT) employing the polarizable continuum model using the integral equation formalism variant (IEFPCM) with water as the model solvent. A transition state corresponding to the transfer of the aldehydic hydrogen of one PGA molecule to the hydroxyl group of the other molecule (i.e., TS1) was observed with a relative energy of 4.77 eV (related to the initial state, expressed as the sum of energies of two PGA molecules, set arbitrarily to 0 eV, min1). Then a minimum with a new C–C bond connecting both PGA molecules (min2) is formed (with a relative energy of 0.58 eV) while a water molecule is released. Subsequently a second transition state (TS2, with an energy of 4.15 eV) leads to the transfer of the hydrogen from the second aldehydic carbon to a hydroxyl group followed by the cleavage of a second water molecule and the final product (i.e., anthrachrysone) is formed with a total relative energy of −0.96 eV, i.e., min3 (see Figure 4). Note that analogous calculations performed in vacuum result in a higher energetic barrier of the whole process represented by the first transition state (TS1) compared to calculations in water (5.13 eV vs. 4.77 eV) suggesting that water facilitates the condensation process.

The peak of ATCS detected in the PGA reaction mixture partially co-elutes with some other compounds providing an identical *m*/*z* value (other tetrahydroxylated anthraquinone (THAQ) isomers). The first eluting, partially resolved isomer was denoted as THAQ1 and is discussed later (Figure 2B, peak at RT 7.67 min). We suggest that these isomers are formed by a keto–enol tautomerization of the ATCS molecule. In fact, trace amounts of these tautomers are also observed in the reconstructed chromatogram of the ATCS standard (with RT range of roughly 7.0–8.6 min). Tautomerization has been already described to occur at hydroxy-substituted anthraquinones (using synthesis, NMR, and computational study) strongly supporting its involvement in the processes observed in our study [35,36].

Interestingly, as mentioned above, the ratio of native and oxidized forms differs among studied anthraquinones. The yield of the oxidized form is higher in ATCS compared to its tautomers. Moreover, this comparison confirms that the peak does not belong to one compound (peak splitting) but instead more isomeric forms are present. The MS/MS spectra of THAQ1 revealed significant differences in the intensity ratio of fragments belonging to the parent and oxidized form, i.e., *m*/*z* 227.0350 and 225.0215, with respect to ATCS (compare spectra at Figure 3A–C). These differences further confirm the above finding.

Moreover, during the independent synthesis of anthrachrysone standard by the mutual reaction of 3,5-dihydroxybenzoic and 3-hydroxybenzoic acids, a fraction eluting after the main deep orange zone containing the main product was collected during preparative chromatographic purification on silica gel (Supplementary Material, Figure S1). It can be assumed that beside reactants this fraction contains also some hydroxylated anthraquinones. This fraction was analyzed by LC/HRMS^2^ and Figure 2C shows the related reconstructed chromatogram at *m*/*z* 271.024. One dominant peak at RT 9.86 min (referred to as tetrahydroxylated anthraquinone isomer 2, THAQ2) can be observed beside traces of ATCS (RT 8.91 min). Interestingly, the peak of the oxidation form of THAQ2 was absent and only a trace amount of the oxidation form corresponding to ATCS can be observed (Figure 2F). On the other hand, except the signal of the oxidation form (i.e., *m*/*z* 269) and its fragment at *m*/*z* 225, other main characteristic fragments of THAQ2 in its MS/MS spectrum correspond with those found in the MS/MS spectrum of ATCS (Figure 3A,B). Additional information for the identification of ATCS (and its isomers) in the PGA reaction mixture is provided by a UV-VIS spectrum that corresponds with the spectrum of the ATCS standard. This compound provides characteristic bands with maxima at 228, 293, 333, and 480 nm. Stodůlková et al. observed a corresponding mass, similar UV-VIS spectra, and fragmentation pattern in the MS/MS spectrum (obtained by FT-ICR-MS) of anthrachrysone (the structure was also confirmed by NMR in this work) [17,18]. Sadilova et al. [5] states that the degradation of anthocyanin dyes results in PGA and phenolic acids (PA) formation. Our research shows that consequent condensation reactions of PGA (and/or PA) and formation of ATCS can indicate degradation of materials rich in anthocyanins (food control, study of organic residues in archaeology, etc.).

To evaluate the suitability of the LC/HRMS^2^ method, we studied repeatability of the retention time and the peak area, linearity, limit of quantification (LOQ), and limit of detection (LOD) for anthrachrysone. It can be seen from the Table 1 that those parameters are acceptable.

Further attention was paid to the effect of pH on the transformation of PGA and the formation of ATCS at elevated temperature (95 °C, for experimental conditions see Section 3.3). In acidic solutions (0.1 mol·L^−1^ formic acid, pH = 3.5, simulating the original acidity of wine), the content of PGA decreased to 12.0% of its original amount after one hour and the content was not changed during further heating (the reaction was monitored for 4 h in total). The appearance of ATCS, however, was not observed. In neutral solution (0.5 mol·L^−1^, sodium phosphate buffer, pH = 7), the content of PGA decreased to 6.5% and 0.4% of its initial amount (100 mg·L^−1^) after one and four hours, respectively. ATCS was formed in detectable amounts (concentration—2.2 mg·L^−1^) immediately after the mixing of PGA with the buffer. After one hour of heating, a concentration of 8.9 mg·L^−1^ was reached showing a strong effect of the temperature on the rate of the reaction. After four hours of heating, the content decreased to 0.7 mg·L^−1^ of ATCS indicating its further decomposition at such high temperature, however, not reversibly back to PGA. This study suggests that an increase of the pH significantly above “natural values” typical for plant food (e.g., pH = 3.5 for wine [37]) causes considerable formation of ATCS (or related isomers) in PGA solutions.

**Figure 5:**
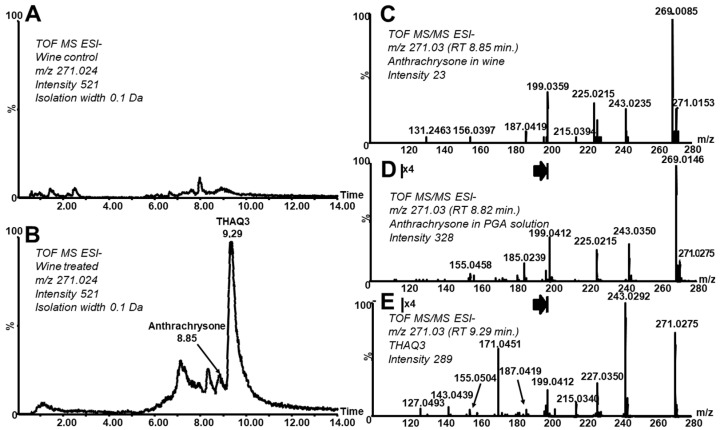
Analysis of wine (reconstructed chromatograms at *m*/*z* 271.0227 in the analysis of untreated red wine (**A**) and red wine treated under BNHC (**B**); MS/MS spectrum of ATCS in treated red wine (**C**); MS/MS spectrum of ATCS rising in heated PGA solution (**D**), for comparison) and THAQ3 isomer eluting at RT 9.29 min (**E**); *m*/*z* range 100–197, denoted by vertical lines and an arrow on the right, was zoomed 4 times).

The processes leading to the formation of hydroxylated anthraquinones via anthocyanin degradation were studied in a sample of red wine. During heating of untreated wine and a wine buffered to pH = 7 formation of ATCS was not reached likely due to (mutual) protecting effects of anthocyanins and other wine components (e.g., via interactions with other polyphenols, tannins, and metals). It was reported that an association with co-pigments provides anthocyanins with protection from the nucleophilic attack of water [2]. However, if the pH value is increased to basic range (pH 8–9) for one hour and then the value is returned to pH 7.0 (“basic–neutral heated conditions”, BNHC) the cleavage of anthocyanins occurred (a decrease in the content of oenin, as the main anthocyanin, roughly to one third of the original amount, as calculated from peak areas in reconstructed chromatogram for *m*/*z* 493.1 was observed) and hydroxylated anthraquinones were formed. PGA (as an intermediate) was transformed during this process. An increase of its amount was not observed under the conditions used (see Supplementary Material S2, reconstructed chromatograms at m/z 155.03 in positive ionization mode), this is in accordance with the study of model solutions. This fact simultaneously means that PGA itself is not generally a suitable long-term marker of anthocyanin-rich foods decomposition. Figure 5 shows differences in intact and treated wine with respect to THAQs (reconstructed chromatogram at 271.03 of the analysis of a control wine and a wine treated under BNHC in the same intensity scale). It can be seen that ATCS and some of its isomers (tautomers) are formed. The MS/MS spectrum averaged over the ATCS peak (Figure 5C) corresponds well with ATCS observed in the PGA model solution and the independently prepared standard solution (Figure 3A,B). The concentration of ATCS in wine treated under BNHC reached a value of 0.01 mg·L^−1^ (determined as 0.5 mg·L^−1^ in a 50× concentrated solution). Another major isomer elutes after the ATCS peak (Figure 5B, RT 9.29, THAQ3). Note that the MS/MS spectrum of this isomer does not contain the signal of the oxidized form (*m*/*z* 269, Figure 5E) and corresponds well with the MS/MS spectrum of THAQ2 (Figure 3D). As already mentioned above, the mutual conversions of isomers of various hydroxylated anthraquinones were described to readily occur [35,36]. A high similarity of already discussed analytical data (i.e., both agreement in mass and fragmentation and virtual absence of the oxidized form in the related spectra of THAQ2 and THAQ3) and the described tautomerization strongly evidences the structural analogy and similarity of THAQ2 and THAQ3. We suggest that THAQ3 could be a tautomeric form of THAQ2 (slightly differing in the distribution of hydroxyl groups over the anthraquinone skeleton). As described in the Introduction section, there are real processes in which anthocyanins are heated and/or exposed to neutral–alkaline solutions/environments causing their decomposition and consequent formation of THAQs. Those processes can occur also when anthocyanin-rich foods stay in contact with clay and/or ceramic material prepared from clay (commonly neutral or slightly alkaline) in archaeological contexts (e.g., a burial site and a settlement). THAQs formation can take place in clay-rich soils due to their high buffering capacity at higher pH values (up to 8.5) [38]. Note that the usage of anthocyanin-rich food is already known as a common part of the diet of Neolithic population [39]. Our experiments show that soil (collected in the archaeologically distinguished and well-known Bull Rock Cave locality [40]) significantly increases the pH of wine solutions. Presence of soil taken from the “Entrance hall” of the cave mixed with solution A (water:wine, 1:1, *v*/*v*) and solution B (water:wine, 9:1, *v*/*v*) increases the pH from 3.2 to 6.4 and from 3.3 to 7.5, respectively. Soil taken from the Madalenian site situated deeper in the cave causes increasing pH of solution A from 3.2 to 5.4 and solution B from 3.3 to 7.2, respectively. Note that the soil from the “Entrance hall” increased the pH value of water (control solution) from 5.5 to 7.9 and the soil from the Madalenian site to 7.6. However, a preliminary analysis of heated wine–soil suspensions did not prove the formation of THAQs. On the basis of model experiments with wine modified to BNHC, we suppose that an increase of pH above 8 can be a crucial inducing step for anthocyanin degradation as a first stage in the formation of anthraquinone derivatives. A more comprehensive research of model and authentic archaeological contexts with respect to those compounds is an objective of our further study.

## 3. Materials and Methods

### 3.1. Chemicals

2,4,6-trihydroxybenzaldehyde (≥97%, Sigma-Aldrich, Merck, Darmstadt, Germany), phosphoric acid (reagent grade, Penta a.s., Czech Republic), sodium hydroxide (reagent grade, Penta a.s., Prague, Czech Republic), *m*-hydroxybenzoic acid (≥99%, Sigma-Aldrich, Merck, Darmstadt, Germany), 3,5-dihydroxybenzoic acid (≥97%, Sigma-Aldrich, Merck, Darmstadt, Germany), glacial acetic acid (≥99% reagent plus, Sigma-Aldrich, Merck, Darmstadt, Germany), acetone (HPLC grade, Fisher-Scientific, Pardubice, Czech Republic) and two-stage purified water (Merck-Millipore, Burlington, MA, USA) were used for all experiments.

### 3.2. Synthesis, Purification and Characterization of 1,3,5,7-Tetrahydroxyanthraquinone

The standard of 1,3,5,7-tetrahydroxyanthraquinone was prepared using a modified method originally described by Briggs and Nicholls in 1951 [8]. Briefly, a solution of *m*-hydroxybenzoic acid and 3,5-dihydroxybenzoic acid was heated with concentrated H_2_SO_4_ for one hour (100 °C). On the next day, the solution was heated (140–150 °C) for 10 min and poured into boiling water. The resulting green precipitate was separated and consequently dissolved in an aqueous solution of NaOH and re-precipitated from a hot solution using glacial CH_3_COOH. The product of this reaction was dissolved in acetone and separated using column preparative chromatography with silica gel 60 (particle size 0.015–0.040 mm, Merck, Germany) as the stationary phase and acetone as the mobile phase (the separation of standard on the silica gel stationary phase is displayed in Supplementary Material, Figure S1). The orange zone was isolated and dried using a fine stream of nitrogen. The peak corresponding to ATCS was dominant in both LC/DAD and LC/MS analyses and its purity, expressed by internal normalization (i.e., ATCS peak area divided by the sum of all peaks present in appropriate chromatograms) reached 96% (from total ion current, TIC, chromatogram, MS detection) and 95% (at λ = 280 nm, UV detection), respectively. The structure of prepared 1,3,5,7-tetrahydroxyanthraquinone was confirmed by nuclear magnetic resonance measurements (Supplementary Material, Figure S3) performed in CD_3_OD solution using VNMRS-400 apparatus (Varian, Palo Alto, USA). The frequencies 399.89 MHz (^1^H) and 100.56 MHz (^13^C) were used for NMR spectra collection. The parameters for ^1^H NMR were as follows: relax. delay 1.000 s, pulse 45.0 degrees, acquire time 2.556 s, width 6410.3 Hz, 512 repetitions, temperature 25 °C, and total time 30 min. The parameters of ^13^C NMR were as follows: relax. delay 1.000 s, pulse 45.0 degrees, acquire time 1.547 s, width 21186.4 Hz, 20,000 repetitions, temperature 25 °C, line broadening 0.5 Hz, and total time 14.1 h.

### 3.3. Preparation of 2,4,6-Trihydroxybenzaldehyde and Red Wine Solutions

PGA (0.5 mg) was dissolved in 5 mL of 0.5 M sodium phosphate buffer (pH 7) or 5 mL of 0.1 M formic acid (pH 3.5) to create 100 mg/L PGA solutions. Aliquots (1 mL) of the solutions were transferred into glass vials (volume 1.5 mL) and closed by crimping caps with polytetrafluorethylene septa. The vials were heated in a drying oven HS32A (Chirana, Brno, Czech Republic) for 0, 1, 4, 8, and 24 h, respectively, at 95 °C. Then the vials were cooled and directly analyzed by LC/HRMS^2^ (the method was identical with that used for THAQs analysis, see Section 3.5).

Fifty milliliters of red wine (Pinot Noir red wine, Paparuda, Cramele Recas, Recas, Romania, vintage 2015) were mixed with 0.260 mL of concentrated phosphoric acid (85 wt.%). The solution was immediately adjusted to pH 7 with 10 M sodium hydroxide using a common pH-meter. This solution was 50× concentrated (evaporated to dryness and reconstituted in 1 mL of mobile phase A), transferred into a glass vial (volume 1.5 mL) and closed by a crimping cap with a PTFE septum. Subsequently, the vial was heated and analyzed in the same way as described above for PGA. In parallel experiments, the pH of the red wine was increased to 8–9 by the addition of the sodium hydroxide solution and heated for one hour (95 °C), then adjusted to pH = 7 with the phosphoric acid and heated for one hour again. Then the solution was concentrated and measured as described above.

### 3.4. Effect of Soil on Acidity and Anthraqtuinone Derivatives Formation in Red Wine

The effect of soil on the pH of wine was studied. Two samples of soil were taken from an archaeologically significant locality—The Bull Rock Cave—the longest cave system in the Moravia region of the Czech Republic. One sample was taken from the Entrance Hall of the cave sanctuary Habrůvka–“Býčí skála” (Hallstatt period, Iron Age, 700 BC–450 AC). The second one was collected approximately 100 m behind the entrance to the cave on the Madalenian site (Magdalenian culture, Upper Paleolithic age, 15,000–10,000 BC). The samples were homogenized and powdered, 10 g of each sample was weighed in a 50 mL glass bottle and mixed with 10 mL of wine solution or distilled water (control). Wine samples were prepared by the dilution of the wine with water in the ratio of 1:1 (*v*/*v*, solution A) and 1:9 (*v*/*v*, solution B). The prepared sample and control suspensions were stirred for 10 min and their pH was measured using a pH-meter. The samples were then heated in a drying oven HS32A (Chirana, Brno, Czech Republic) for 24 h at 95 °C and analyzed by LC/HRMS^2^.

### 3.5. High Performance Liquid Chromatography/High Resolution Tandem Mass Spectrometry

An Acquity I-Class UPLC chromatographic system connected to Synapt G1 high-resolution tandem mass spectrometer (both Waters, Milford, CT, USA) was used for analyses of PGA solutions and wine samples. The separation was performed on an Ascentis Express C18 column (100 × 2.1 mm^2^, d_p_ = 2.7 µm, porous shell layer 0.5 µm, Sigma-Aldrich). Binary gradient elution using mobile phases A: 0.1% HCOOH in water (*v*/*v*) and B: 0.1% HCOOH in methanol (*v*/*v*) was used. A following profile of gradient was used: 0–4.28 min 20% B, 4.28–7.85 min 20–60% B, 7.85–13.56 min 60% B, 13.56–14.99 min 60–20% B, and 14.99–22.13 min 20% B at flow rate 0.35 mL·min^−1^. The injection volume was 10 µL.

The parameters of MS measurements were as follows: spray voltage −3.0 kV (negative ion mode), source temperature 120 °C, sampling cone 40 V, desolvation temperature 250 °C, cone gas flow rate 0 L·h^−1^, and desolvation gas flow rate 600 L·h^−1^. Positive ionization mode was used for oenin and PGA analysis in the red wine samples. Parameters of MS method (except for spray voltage, +2.5 kV) were the same as in negative ion mode. Fragmentation was performed in the Trap collision cell using a collision energy of 20 eV. In the Transfer collision cell, a collision energy of 4 eV was applied.

### 3.6. Quantum Calculations of PGA Condensation Reaction to Anthrachrysone

The structures of PGA and ATCS were modelled in Avogadro software (version 1.1.0) and preoptimized using MMFF94 force field. Subsequently, an optimization using B3LYP/6-31G* functional/basis set combination was performed using Gaussian 09 (Gaussian, Inc., Wallingford, CT, USA). Frequency calculation was performed and the results enabled us to identify minima (i.e., structures with the absence of imaginary frequencies) and transition states (i.e., structures with exactly one imaginary frequency). The energies of target structures in solution (expressed as Gibbs free energies) were obtained using Tomasi’s polarized continuum model (PCM) as implemented in Gaussian 09. Default values in Hartrees were recalculated to eV using the following formula: 1 Ha = −27.211, 386,245,988 eV [41]. For the quantum calculations, we used a cluster consisting of 2 computers, each equipped with 2 Intel Xeon E5 2420 1.9 GHz, 7.2 GT 15 MB cache, 6-core processors and 6 GB 1333 MHz DDR3 RAM.

## 4. Conclusions

The process of transformation of anthocyanins via their decomposition to phloroglucinaldehyde and its consequent condensation reactions were studied by LC/HRMS^2^. First, the formation of 1,3,5,7-tetrahydroxyanthraquinone and its isomers was proven in a heated neutral aqueous solution of phloroglucinaldehyde. Consequently, LC/HRMS^2^ experiments proved the presence of tetrahydroxyanthraquinones (THAQs) in red wine after an adjustment to a neutral–slightly alkaline pH region. The concentration of 1,3,5,7-tetrahydroxyanthraquinone in a treated wine reached 0.01 mg·L^−1^. The quantum calculations performed enabled exploration of the proposed condensation mechanism and confirmed the possibility of formation of anthrachrysone from phloroglucinaldehyde. We suggest that detection of tetrahydroxyanthraquinone isomers (together with evaluation of common phenolics profile) in anthocyanin-rich food can be applied to reveal processes involving high temperature/reduction of acidity during food production (either wanted or undesirable; as a part of food quality and authenticity control) or to indicate anthocyanin-rich fruit/related food remains (e.g., in archaeological findings). This is the objective of our upcoming research.

## Figures and Tables

**Table 1:** Parameters for quantification of 1,3,5,7-tetrahydroxyanthraquinone.

Parameter	
Concentration range (mg·L^−1^)	0.5–20.0
Regression equation	y = 25.9x + 80.7
R^2^	0.95
RT CV (%) ^a^	0.2
Area CV (%) ^b^	1.2
LOQ (mg·L^−1^)	0.4
LOD (mg·L^−1^)	0.2

^a^—calculated from 9 analyses of antrachrysone standard; ^b^—calculated for concentration level 5.0 mg·L^−1^; CV—coefficient of variation; LOQ and LOD—limit of quantification and detection, respectively.

## Supplementary Materials

The following are available online, **Figure S1:** Separation of synthesized 1,3,5,7-tetrahydroxyanthraquinone on silicagel stationary phase; **Figure S2:** Reconstructed chromatograms of compounds with *m*/*z* 155.03 for PGA standard solution (A) and red wine control (untreated, B) and red wine treated under BHNC (C); **Figure S3:** NMR spectra of synthesized standard of 1,3,5,7-tetrahydroxyanthraquinone. A—^1^H spectrum; B—^13^C spectrum; C—HSCQ spectrum and D—HMBC spectrum.
