# Supplementary material for: Study of Tetrahydroxylated Anthraquinones—Potential Tool to Assess Degradation of Anthocyanins Rich Food
Source: Molecules. 2020 Dec 22;26(1):2. doi: 10.3390/molecules26010002 (PMC7792584; doi:10.3390/molecules26010002)
Supplement: Supplementary file 1 [file molecules-26-00002-s001.pdf]

Supplementary Material

Study of tetrahydroxylated anthraquinones – potential tool to assess degradation of anthocyanins rich food

Lukáš Kučera[1], Ondřej Kurka[1], Martin Golec[2] and Petr Bednář[1*]
[1] Department of Analytical Chemistry, Faculty of Science, Palacký University, 17. listopadu 12, 779 00, Olomouc, Czech Republic; lukas.kucera@upol.cz, ondrej.kurka@upol.cz
[2] Department of History, Faculty of Arts, Palacký University, Křížkovského 10, 779 00, Olomouc, Czech Republic; martin.golec@upol.cz

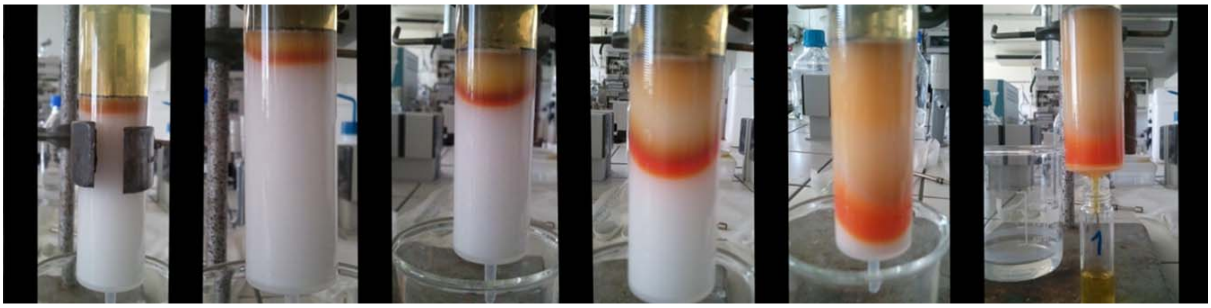

Figure S1. Separation of synthesized 1,3,5,7-tetrahydroxyanthraquinone on silicagel stationary phase

* Corresponding author. Department of Analytical Chemistry, Faculty of Science, Palacký University, 17.listopadu 12, 779 00, Olomouc, Czech Republic
Tel.: +420585634403, E-mail address: petr.bednar@upol.cz

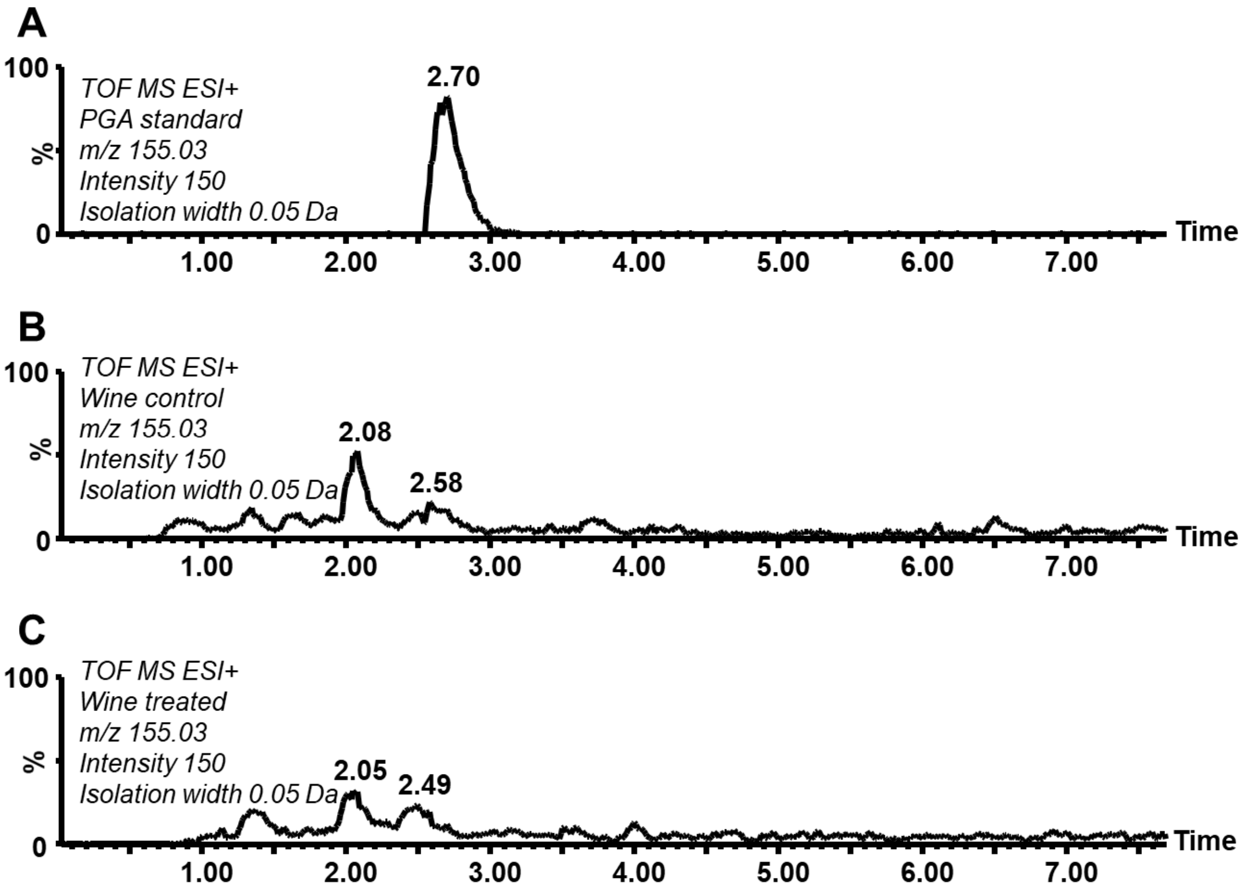

Figure S2. Reconstructed chromatograms of compounds with m/z 155.03 for PGA standard solution (**A**) and red wine control (untreated, **B**) and red wine treated under BNHC (**C**).

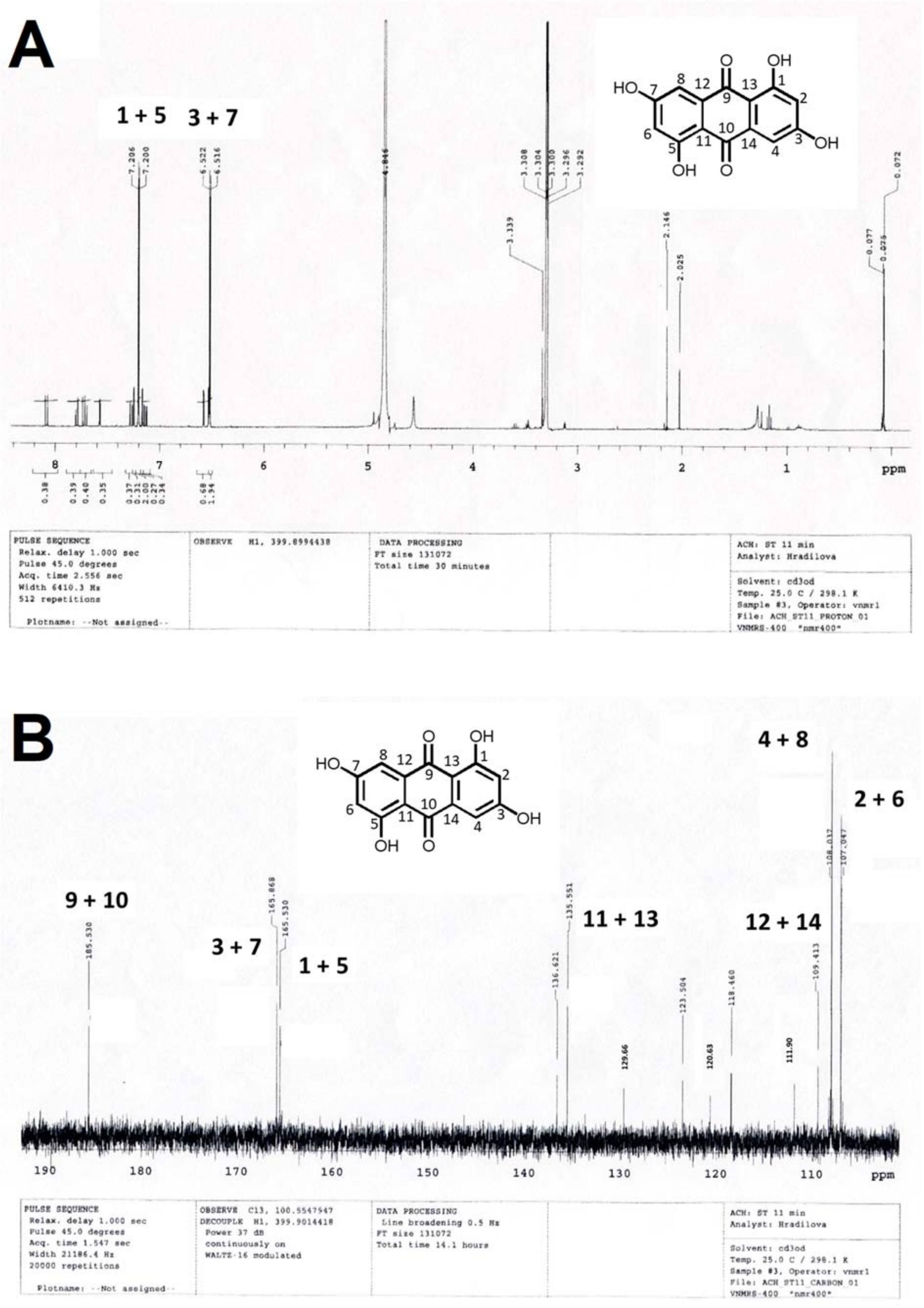
A
1 + 5
3 + 7
PULSE SEQUENCE
Relax. delay 1.000 sec
Pulse 45.0 degrees
Acq. time 2.556 sec
Width 6410.3 Hz
512 repetitions
Plotname: --Not assigned--
OBSERVE H1, 399.8994438
DATA PROCESSING
FT size 131072
Total time 30 minutes
ACH: ST 11 min
Analyst: Hradilova
Solvent: cd3od
Temp. 25.0 C / 298.1 K
Sample #3, Operator: vnmrl
File: ACH_ST11_PROTON_01
VNMRS-400 "nmr400"
ppm
B
9 + 10
3 + 7
1 + 5
11 + 13
12 + 14
4 + 8
2 + 6
129.66
120.63
111.90
ppm
PULSE SEQUENCE
Relax. delay 1.000 sec
Pulse 45.0 degrees
Acq. time 1.547 sec
Width 21186.4 Hz
20000 repetitions
Plotname: --Not assigned--
OBSERVE C13, 100.5547947
DECOUPLE H1, 399.9014418
Power 37 dB
continuously on
WALTZ-16 modulated
DATA PROCESSING
Line broadening 0.5 Hz
FT size 131072
Total time 14.1 hours
ACH: ST 11 min
Analyst: Hradilova
Solvent: cd3od
Temp. 25.0 C / 298.1 K
Sample #3, Operator: vnmrl
File: ACH_ST11_CARBON_01
VNMRS-400 "nmr400"

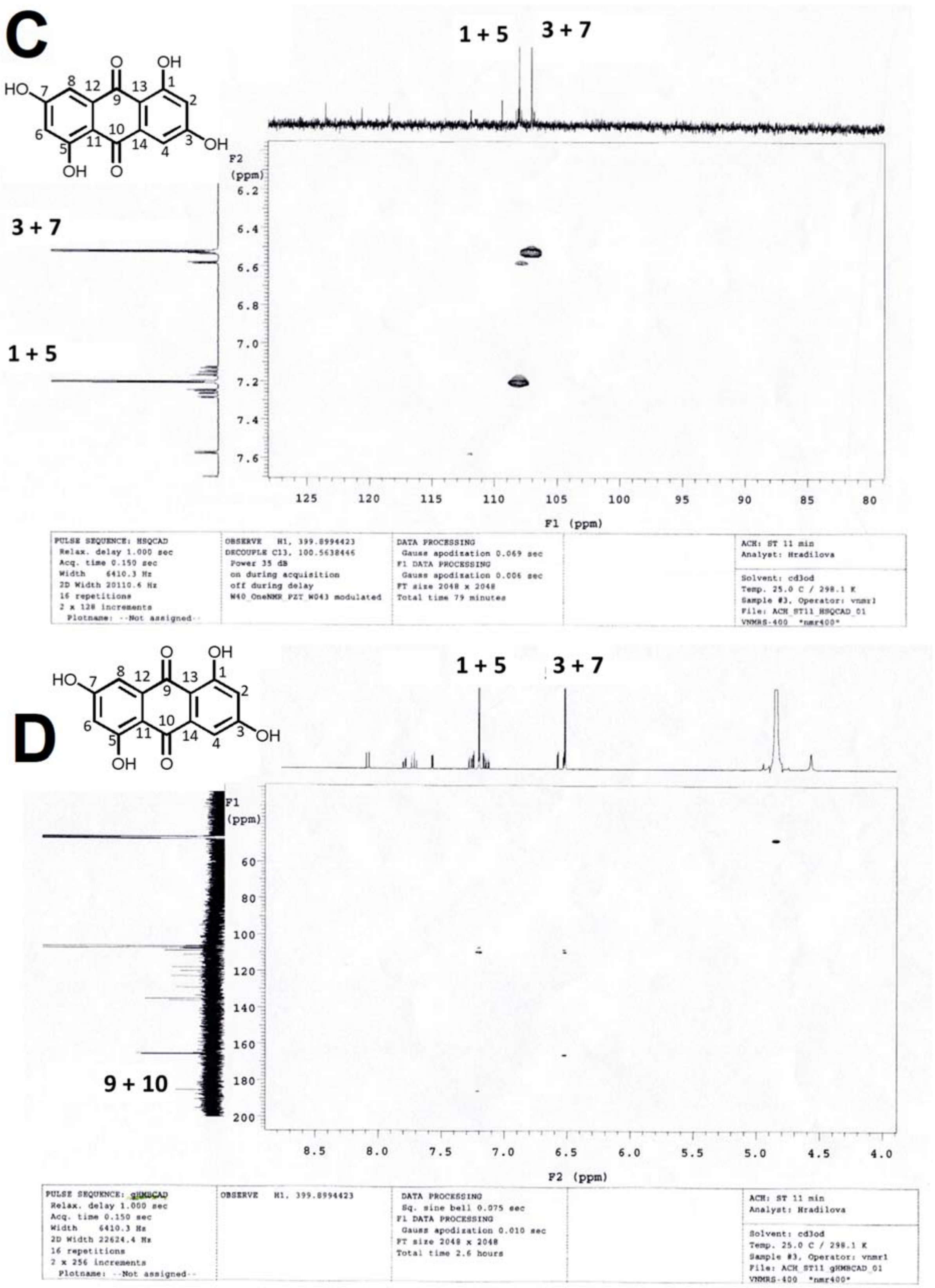

Figure S3. NMR spectra of synthesized standard of 1,3,5,7-tetrahydroxyanthraquinone. **A** – ^{1}H spectrum; **B** – ^{13}C spectrum; **C** - HSCQ spectrum and **D** – HMBC spectrum.
